# Anti-neoplastic activity of celastrol in experimentally-induced mammary adenocarcinoma in mice: targeting wnt/β-catenin signaling pathway

**DOI:** 10.1007/s00210-025-04148-1

**Published:** 2025-04-28

**Authors:** Mohamed M. Salama, Randa A. Zaghloul, Rania M. Khalil, Mamdouh M. El-Shishtawy

**Affiliations:** 1 Department of Biochemistry, Faculty of Pharmacy, Delta University for Science and Technology, Gamasa, 35712 Egypt; 2 Department of Biochemistry, Faculty of Pharmacy, Mansoura University, Mansoura, 35516 Egypt

**Keywords:** Ehrlich solid carcinoma, Celastrol, Doxorubicin, β-catenin, Cyclin-D1, Surviving

## Abstract

Natural bioactive compounds with anti-neoplastic effects, such as celastrol (CLS), have attracted considerable interest in recent years. The present study aimed to investigate the effect of CLS on wnt/β-catenin signaling, and its potential combination with doxorubicin (Dox) to enhance chemotherapeutic effects. After intramuscular inoculation of Ehrlich tumor cells, tumor-bearing mice received CLS (2 mg/kg**,** i.p), Dox (5 mg/kg, once/week, i.p), and their combination for 21 days. Treatment with CLS showed showing antioxidant and anti-inflammatory, as evidenced by a significant increase in glutathione content and a significant decrease in the malondialdehyde, interleukin 6, and interleukin 1β concentrations. CLS also inhibited VEGF-mediated angiogenesis. The current study revealed that CLS downregulated *β‐catenin* gene expression with subsequent downstream target genes, such as *cyclin-D1*, and *survivin*, which dampens tumor cell proliferation and triggers cell cycle arrest as well as induces apoptosis as indicated by the increased expression of p53, caspase‐3. The current study concludes that CLS exerted its anti-neoplastic activity by suppressing the wnt/β-catenin signaling pathway, and opens a new perspective for combining CLS with Dox to enhance its chemotherapeutic effects and reduce the oxidative imbalance and inflammatory responses associated with Dox treatment.

## Introduction

Cancer remains one of the biggest global health issues today due to the disease’s complexity and mutability. Moreover, despite the major advances in understanding its etiology and pathogenesis to improve prevention and treatment methods, the disease remains a major cause of natural death worldwide (Biemar and Foti 2013). There are several *in-vivo* experimental models of cancer including Ehrlich carcinoma, a neoplasm of epithelial malignant origin, corresponding to murine mammary adenocarcinoma, which is an aggressive and fast-growing carcinoma able to develop both in the ascitic and the solid form depending on whether inoculated intraperitoneally or subcutaneously, respectively, and frequently utilized as a tumor model to evaluate the tumor pathogenesis and investigation of anti-neoplastic activity of various products (Aldubayan et al. 2019; Feitosa et al. 2020).

The identification of new bioactive compounds derived from medicinal plants with significant therapeutic properties has attracted considerable interest in recent years. One such promising natural bioactive compound derived from a traditional Chinese plant, Tripterygium wilfordii Hook F, is celastrol (CLS), which has been found to provide substantial anti-neoplastic effects against sundry tumors (Ng et al. 2019; Shi et al. 2020). These anti-neoplastic properties can be attributed to inducing apoptosis along with suppressing tumor proliferation, angiogenesis, and migration by targeting multiple signaling pathways. However, the molecular mechanisms of CLS in the treatment of cancer and inhibiting tumor cell proliferation need further investigation (Lim et al. 2021).

The Wnt/β-catenin pathway is a conserved signaling axis regulating several physiological processes, such as cell proliferation, differentiation, adult tissue homeostasis, and apoptosis (Steinhart and Angers 2018). Increasing evidence indicates that dysregulation of that pathway and its transcription factor β-catenin, which is the central mediator of the Wnt/β-catenin signaling, contributes to the development and progression of some solid tumors and hematological malignancies (Zhang and Wang 2020). β‐Catenin can trigger the transcription of several downstream target genes, such as cellular myelocytomatosis oncogene (*c-Myc*), *cyclin-D1*, and *survivin*, involved in inhibiting apoptosis and driving cell cycle progression and eventually cell proliferation (Bu et al. 2019; Ji et al. 2022).

The recent study aimed to determine whether CLS could suppress the proliferation of Ehrlich cells by inhibiting the wnt/β-catenin signaling pathway. Also, combining CLS with chemotherapies, like doxorubicin (Dox), for improved efficacy and minimizing ominous side effects, mediated mainly by oxidative stress and inflammatory response (Özturk et al. 2024), remains a topic of active investigation.

## Material and methods

### Animals

Female Swiss albino mice (Mus musculus species) were purchased from Theodor Bilharz Research Institute, Cairo (Egypt), with a body weight of 20–25 g and kept in steel mesh cages. Male mice were excluded because of their poor tumor growth (Vincent and Nicholls 1967). All animals were housed in a standard animal facility under controlled environmental conditions at room temperature 22 ± 2 °C, 45–55% humidity, and a 12-h light–dark cycle. Mice were fed standard pellet chow (23% protein and 4% fat) and allowed free access to tap water. Animals were kept under the same circumstances for 7 days before the experiment for acclimatization. The animal care and experiments described in this study comply with the ethical principles and guidelines for the care and use of laboratory animals adopted by the"Research Ethics Committee"of Faculty of Pharmacy, Mansoura University, Mansoura, Egypt with approval number (2019–143), which follow the ARRIVE guidelines and the National Research Council’s Guide for the Care and Use of Laboratory Animals (Committee for the Update of the Guide for the Care and Use of Laboratory Animals, 2011).

### Drugs and chemicals

CLS was purchased from Sigma-Aldrich “St. Louis, MO, USA” and dissolved in dimethyl sulfoxide (DMSO) at 10 mg/ml and then diluted with normal saline to the final concentrations (1% DMSO) before administration. Dox under the brand name of Adriablastina® vial was obtained from Pharmacia Italia S.P.A. “Nerviano, Italy”. All other used reagents and chemicals were of diagnostic and analytical grade.

### Cell line

Ehrlich ascites carcinoma (EAC) is of a mammary origin and functioned as the original tumor from which Ehrlich solid tumor was obtained. The EAC cell line (RRID: CVCL_3873) was obtained from the Pharmacology and Experimental Oncology Unit of the National Cancer Institute, Cairo University, Cairo, Egypt. The cells were maintained in ascetic form through transplantation of 2.5 × 10^6^ tumor cells into the peritoneal cavity of the mice and allowed to multiply. The ascetic fluid containing Ehrlich tumor cells was developed within 10 days and then collected followed by dilution with normal saline (1:10) and counting via a Hemocytometer (Sigma-Aldrich, St. Louis, MO, USA). The viability of the cells used is set to be not less than 95% as confirmed by the trypan blue (Sigma-Aldrich, St. Louis, MO, USA) exclusion assay (Louis and Siegel 2011).

### Experimental design

Following a period of 7 days for acclimatization, a solid tumor was induced by intramuscular inoculation of 0.2 ml of ascetic fluid, containing approximately 2 × 10^6^ EAC cells, in the right thigh of the hind limb of each mouse (Adami et al. 2020). When all solid tumors reach a size of 50–100 mm^3^, animals were randomly divided into four groups (*n* = 10) as followings:

- **Untreated control group:** Mice received an intraperitoneal (i.p) injection of 1% DMSO diluted with normal saline daily for 21 days.
- **CLS-treated group:** Mice received CLS (2 mg/kg**,** i.p) daily for 21 days (Lin et al. 2015; Yu et al. 2018).
- **Dox-treated group:** Mice received Dox (5 mg/kg**,** i.p) once a week for 21 days **(**Rana et al. 2013**)**.
- **CLS & Dox-treated group:** Mice received Dox (5 mg/kg**,** i.p) once a week and CLS (2 mg/kg**,** i.p) daily for 21 days.

### Tumor volume and growth inhibition rate

The tumor mass was measured five days after the tumor cell injection and then every five days for a total of 21 days. The volume of the solid tumor was measured using a Vernier caliper (Tricle Brand, Shanghai, China) according to the following formula (Psurski et al. 2019):

$$\text{Tumor volume }\left({\text{mm}}^{3}\right)=0.5\;(\text{Length}\times {\text{Width}}^{2})$$

While tumor inhibition rate was calculated using the following formula (Facchini et al. 2014):

$$\mathrm{Tumor}\;\mathrm{growth}\;\mathrm{inhibition}\;(\%)\;=\;\lbrack(\mathrm{Mean}\;\mathrm{tumor}\;\mathrm{volume}\;\mathrm{of}\;\mathrm{the}\;\mathrm{untreated}\;\mathrm{control}\;\mathrm{group}\;-\;\mathrm{Mean}\;\mathrm{tumor}\;\mathrm{volume}\;\mathrm{of}\;\mathrm{the}\;\mathrm{treated}\;\mathrm{group})/\;\mathrm{Mean}\;\mathrm{tumor}\;\mathrm{volume}\;\mathrm{of}\;\mathrm{intreated}\;\mathrm{control}\;\mathrm{group}\rbrack\;\times\;100$$

### Tissue sampling

After 21 days of treatment, mice were sacrificed using isoflurane in chamber induction according to IACUC guidelines and the tumor mass was harvested, weighed, and separated into three portions. The first part was fixed in 10% neutral buffered formalin (El-Nasr Chemicals Co., Cairo, Egypt) to be embedded in paraffin wax for histopathological and immunohistochemical examination. The second part was homogenized (10% w/v) in ice-cold phosphate-buffered saline (PBS) (0.02 M, pH 7.4) using a tissue homogenizer (Heidolph Silent-Crusher M, Schwabach, Germany). The homogenates were then centrifuged at 5000 rpm for 5 min at 4 °C, and the resulting supernatants were stored at − 80 °C for the subsequent biochemical analysis. The last part was preserved in RNA*later*® (Qiagen, Hilden, Germany, Cat. #76104), incubated overnight at 2–8 °C, and then stored at − 80 °C for further RNA extraction.

### Histopathological examination

Tissue specimens collected from the tumor-bearing thigh of different groups were excised and fixed in 10% neutral buffered formalin (pH 7.4), and then the samples were processed through dehydration in ascending concentrations of ethanol, clearance in xylene, and embedding in paraffin. From each paraffin block, 4 μm thick sections were cut and stained by hematoxylin and eosin (H&E) for light microscopic evaluation. All sections were blindly photographed and examined by an independent pathologist.

### Immunohistochemical (IHC) analysis

P53 and caspase- 3 markers were measured immunohistochemically using the BioModule™ IHC staining Kit (Invitrogen™, Carlsbad, CA, USA, Cat. #WFGE11), according to the manufacturer's protocol. Paraffin sections were cleared in xylene, rehydrated, and treated with Peroxo-Block™, a specific inhibitor of endogenous peroxidase activity, to efficiently remove endogenous peroxidase activity followed by heat-induced epitope retrieval through immersing in diluted citrate buffer (pH 6.0) and boiling the solution for 15 min. The sections were incubated with p53 polyclonal antibody (ABclonal, Woburn, MA, USA, Cat. #A3185; 1:100 dilution) or caspase- 3 monoclonal antibody (GeneTex, Irvine, CA, USA, Cat. #GTX30246; 1:200 dilution). After washing with PBS, the sections were incubated with a horseradish peroxidase enzyme (HRP) polymer conjugate, visualized with diaminobenzidine (DAB) chromogen, and finally counterstained with Mayer's hematoxylin. Slides were photographed using an Olympus® digital camera that was attached to an Olympus® microscope (Shinjuku, Tokyo, Japan), and then the brown color density which represents the p53 or caspase- 3 protein expression was analyzed using ImageJ software version 1.2.4, RRID: SCR_003070 (National Institutes of Health, Bethesda, MD, USA).

### Assessment of oxidative stress and inflammatory biomarkers

Following the given instructions, malondialdehyde (MDA) and reduced glutathione (GSH) were measured spectrophotometrically in the tumor tissue homogenate using commercially available kits (Bio Diagnostic, Giza, Egypt). Besides, the concentrations of interleukin 6 (IL- 6) and interleukin 1β (IL- 1β) in tumor homogenate were determined by the ELISA (enzyme-linked immunosorbent assay) technique using commercially available kits obtained from Aviva Systems Biology (San Diego, CA, USA) as per the manufacturer's instructions.

### Quantitative real-time polymerase chain reaction (qRT-PCR)

Total RNA was extracted from the tumor-bearing thigh collected from different mice groups using Qiagen RNeasy® Mini Kit (Qiagen, Hilden, Germany, Cat. #74104) in an RNase-free environment, following the manufacturer's protocol. The RNA concentration and purity were measured spectrophotometrically (260, 260/280 nm ratio, respectively) using the NanoPhotometer® P330 (Implen, Schatzbogen, München, Germany). A total of 1 μg of purified RNA was reverse transcribed into complementary DNA (cDNA) using RevertAid First Strand cDNA Synthesis Kit (Thermo Fisher Scientific Inc., Vantaa, Finland, Cat. #K1622). *VEGF* (vascular epithelial growth factor), *β-catenin*, *cyclin-D1*, and *survivin* mRNA levels were measured using Bioline SensiFAST™ SYBR® No-ROX Kit (Meridian Bioscience™, Memphis, TN, USA, Cat. #BIO- 65053) and PikoReal 96™ Real-Time PCR System ***(***Thermo Fisher Scientific Inc., Vantaa, Finland). Meanwhile, mouse *GAPDH* (glyceraldehyde 3-phosphate dehydrogenase) was used as a housekeeping gene and an internal reference control. The sequences of forward and reverse primers are illustrated in Table 1. Relative expression of the studied genes was calculated using the 2^−ΔΔCT^ method (Livak and Schmittgen 2001), normalized with respect to *GAPDH* mRNA, and relative to a calibrator sample. Untreated control samples were used as calibrators.

**Table 1:** The primer sequences used for quantitative real-time PCR

Primer		Sequence	NCBI reference sequence	Amplificationsize
*VEGF*	F	5’-CACGACAGAAGGAGAGCAGAAG- 3’	NM_001025250.3	82
	R	5’-CTCAATCGGACGGCAGTAGC- 3’		
*β-Catenin*	F	5’-GTTCGCCTTCATTATGGACTGCC- 3’	NM_007614.3	146
	R	5’-ATAGCACCCTGTTCCCGCAAAG- 3’		
*Cyclin-D1*	F	5’-AGAAGTGCGAAGAGGAGGTC- 3’	NM_001379248.1	157
	R	5’-TTCTCGGCAGTCAAGGGAAT- 3’		
*Survivin*	F	5’-GAATCCTGCGTTTGAGTCGT- 3’	NM_001012273.1	207
	R	5’-AATCAGGCTCGTTCTCGGTA- 3’		
*GAPDH*	F	5’-ATGGTGAAGGTCGGTGTGAAC- 3’	NM_008084.3	251
	R	5’-TTGATGTTAGTGGGGTCTCGC- 3’		

*VEGF*: vascular endothelial growth factor; *GAPDH*: Glyceraldehyde 3-phosphate dehydrogenase; F: forward; R: reverse

The primers used in this study were designed to be 20 to 23 bases long with a G/C content between 50–60%, which is optimal for qRT-PCR. Primer design was carried out using Primer-BLAST (NCBI, Bethesda, MD, USA) after obtaining the gene-specific sequences from PubMed GenBank (NCBI, Bethesda, MD, USA). NetPrimer software (PREMIER Biosoft, San Francisco, CA, USA) was used to analyze the primers and identify their oligonucleotide properties. All primers were evaluated for secondary structure formation and showed a rating of 100% without any significant secondary structures. Finally, each primer was subjected to BLAST analysis on NCBI/BLAST (Bethesda, MD, USA) to confirm its specificity to the target gene.

### Statistical analysis

Statistical analysis was performed using GraphPad Prism software version 6 (La Jolla, CA, USA, RRID: SCR_002798). The dissimilarities between the groups were evaluated by one-way ANOVA followed by Tukey's post-hoc test. The data are expressed as the Mean ± SD. The differences between the groups were considered statistically significant at a *p*-value < 0.05.

## Results

### Growth suppressive effect of CLS, Dox, and their combination on ESC-bearing mice

**Fig. 1:**
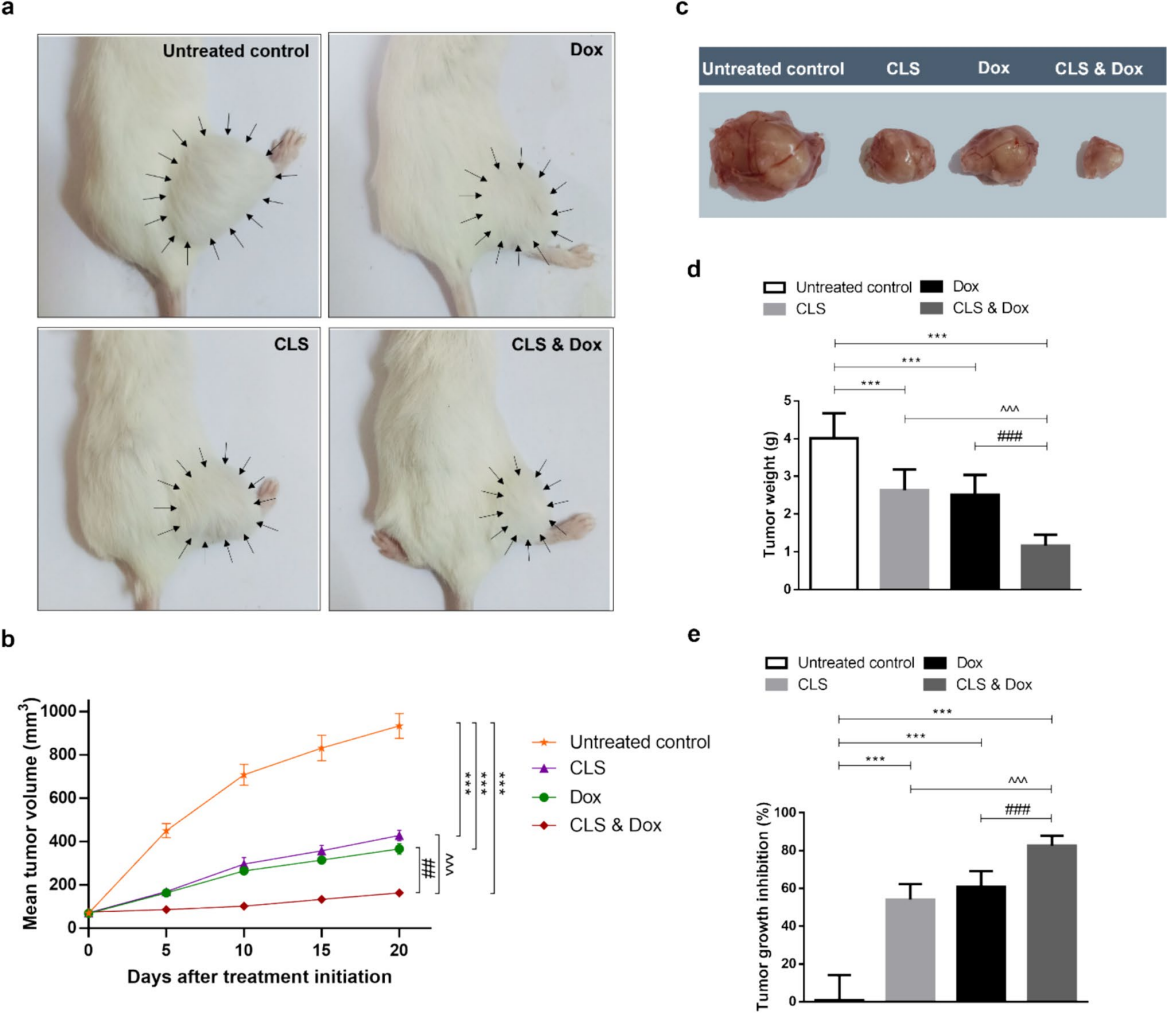
Growth suppressive effect of celastrol (CLS) alone or in combination with doxorubicin (Dox) on Ehrlich solid carcinoma (ESC)-bearing mice. **a** Photographs of representative ESC mouse models from each treatment group at the last day of treatment. Arrows indicate the margins of the tumors. **b** Tumor volume (mm^3^) at each time point after the onset of treatment. **c** The gross appearance of excised tumors from ESC–bearing mice and different treatment groups on the last day of treatment. All tumor images were taken at the same magnification power, zooming, and distance from the camera. **d** Excised tumor weight (g) on the last day of treatment. **e** Percentage of tumor growth inhibition compared to untreated ESC-bearing mice. Results are expressed as Mean ± SD (*n* = 10). Statistical analysis was performed using one-way ANOVA, followed by Tukey's post-hoc test. *** significant at *p* < 0.001 vs. untreated control group; ### significant at *p* < 0.001 vs. Dox-treated group; ^^^ significant at *p* < 0.001 vs. CLS-treated group

The growth-suppressive effect of CLS, Dox, and their combination were evaluated by assessing the mean tumor volume (mm^3^) of the ESC in the right thigh (Fig. 1a, b) and the weight (g) of the solid tumor (Fig. 1c, d), as well as the tumor growth inhibition percentage (Fig. 1e). After 21 days of initial treatment, all treated ESC mice showed a significant decrease in the mean tumor volume and weight compared to the untreated tumor‐bearing mice. The most significant decrease was observed in the combined CLS and Dox-treated group, with either CLS or Dox mono-treatment groups showing nearly the same results. This represents a regression in the tumor growth by 54.14%, 62.47%, and 82.52% for CLS, Dox, and their combination, respectively compared to the untreated ESC-bearing mice. A significant difference was observed in the combined CLS and Dox-treated group regarding the percentage of tumor growth inhibition when compared with either CLS (*p* < 0.001) or Dox (*p* < 0.001) treated group.

### Effect of treatment with CLS, Dox, and their combination on the tumor tissue investigated by the histopathological examination

**Fig. 2:**
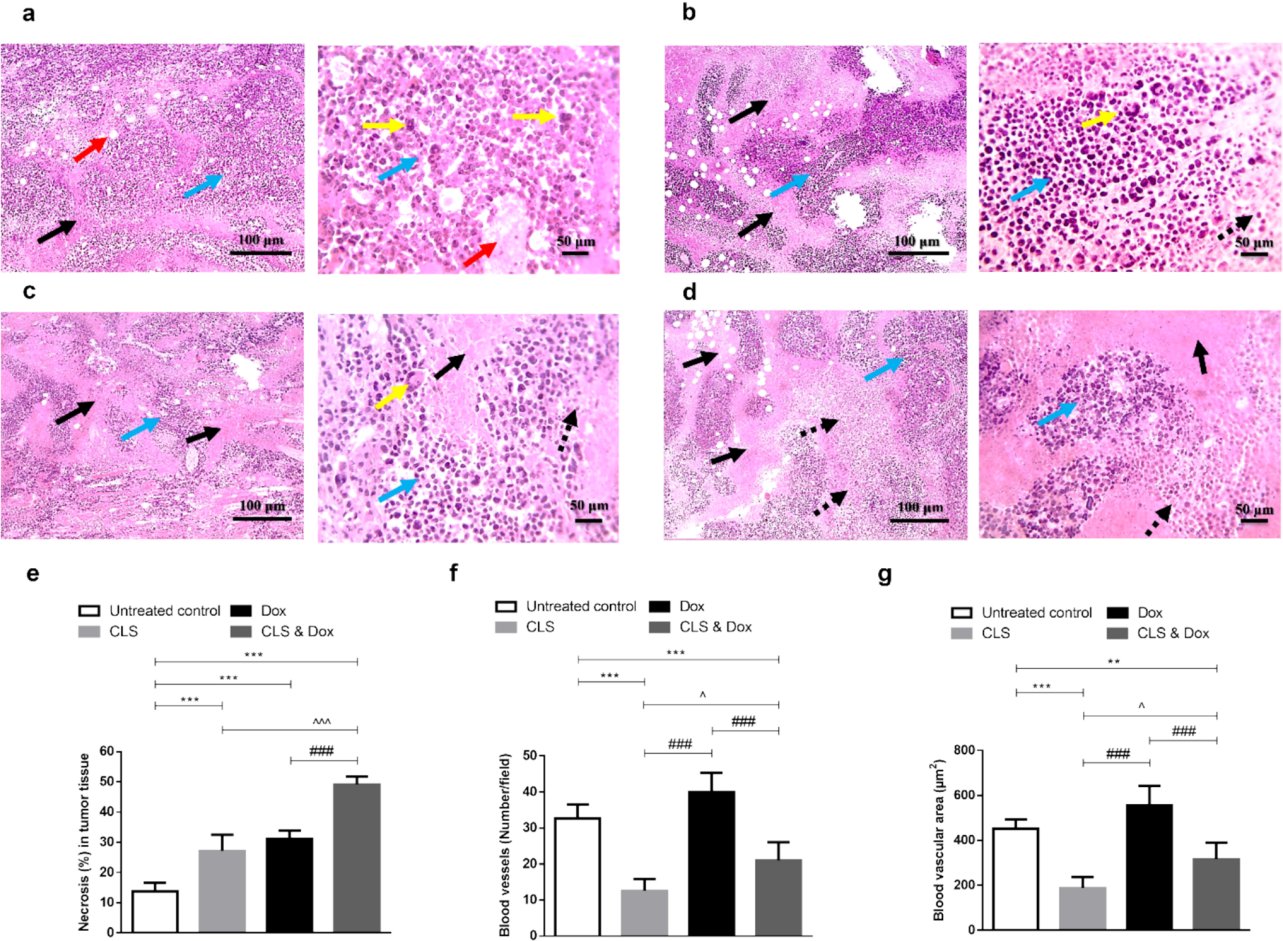
Photomicrograph of tumor sections stained with hematoxylin and eosin (H&E) from Ehrlich solid carcinoma (ESC)-bearing mice. **a** Untreated control group. **b** Celastrol (CLS)-treated group. **c** Doxorubicin (Dox)-treated group. **d** CLS & Dox-treated group. Black arrows indicate necrotic areas, blue arrows indicate viable areas, red arrows indicate newly formed blood capillaries, dashed black arrows indicate ghost cells, and yellow arrows indicate tumor giant cells. Low-magnification (× 100) scale bars = 100 μm and high-magnification (× 400) scale bars = 50 μm. **e** The percentage of necrotic areas in tumor tissue. **f** Number of blood vessels (number/field). **g** Tumor vascular area (μm^2^). Data are expressed as Mean ± SD (*n* = 10). Statistical analysis was performed using one-way ANOVA, followed by Tukey's post-hoc test. ***, ** significant at *p* < 0.001, *p* < 0.01, respectively vs. untreated control group; ### significant at *p* < 0.001 vs. Dox-treated group; ^^^, ^ significant at *p* < 0.001, *p* < 0.05, respectively vs. CLS-treated group

The tumor tissue sections of the untreated control mice have shown multiple large-sized viable tumor areas consisting of pleomorphic ESC cells with numerous tumor giant cells, surrounded by minimal necrosis with numerous newly formed blood capillaries within the tumor tissue (Fig. 2a). Treatment with either CLS (Fig. 2b) or Dox (Fig. 2c) suppressed the tumor growth as indicated by the increased size of necrotic areas and several ghost cells with fewer numbers of neoplastic and tumor giant cells in the viable areas. Likewise, the co-treatment group with CLS and Dox showed a more pronounced reduction in tumor growth than in the mono-treatment groups (Fig. 2d).

The histological scoring revealed a marked increase in the percentage of necrotic areas in CLS or Dox-treated mice compared to the control tumor-bearing group (*p* < 0.001 and < 0.001, respectively) (Fig. 2e). The increase of necrotic areas was more pronounced in the combined CLS and Dox-treated mice when compared to the untreated control, CLS-treated, and Dox-treated groups (*p* < 0.001, < 0.001, and < 0.001, respectively). Moreover, a significant decrease in the number of tumor vessels (Fig. 2f) and tumor vascular area (Fig. 2g) was observed in CLS-treated groups either alone (*p* < 0.001 and < 0.001, respectively) or in combination with Dox (*p* < 0.001 and < 0.01, respectively) compared to the untreated control group. In contrast, the Dox-treated group exerted a slight non-significance increase in tumor vasculature compared to the untreated control group.

### Effect of treatment with CLS, Dox, and their combination on oxidative stress and inflammatory parameters, as well as the gene expression of the angiogenic marker, *VEGF*, in the tumor tissue

**Fig. 3:**
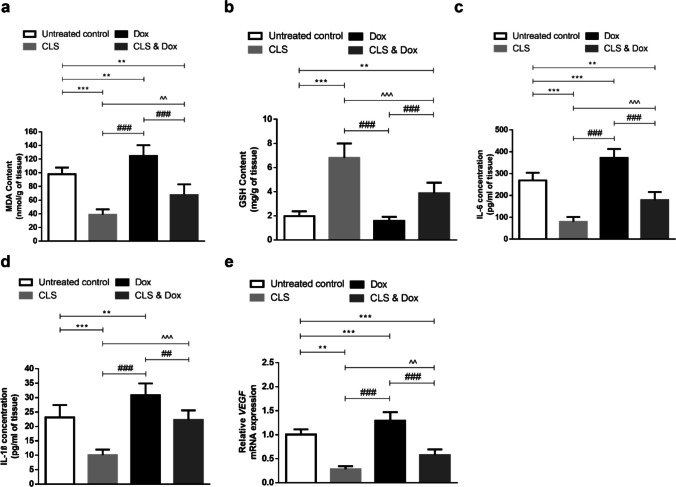
Effect of celastrol (CLS) alone or in combination with doxorubicin (Dox) on oxidative status and inflammatory parameters, as well as the angiogenic *VEGF* gene expression in tumor tissue. **a** Malondialdehyde (MDA). **b** Glutathione (GSH). **c** Interleukin 6 (IL- 6). **d** Interleukin 1β (IL- 1β). **e** vascular endothelial growth factor (*VEGF*). Results are expressed as Mean ± SD (*n* = 10). Statistical analysis was performed using one-way ANOVA, followed by Tukey's post-hoc test. ***, ** significant at *p* < 0.001, *p* < 0.01, respectively vs. untreated control group; ###, ## significant at *p* < 0.001, *p* < 0.01, respectively vs. Dox-treated group; ^^^, ^^ significant at *p* < 0.001, *p* < 0.01, respectively vs. CLS-treated group

Tumor lipid peroxidation marker, MDA, and the antioxidant molecule, GSH, of the different experimental groups are shown in Fig. (3a, b). Unfortunately, treatment with Dox on its own exhibited a significant increase in MDA content as compared to the untreated tumor‐bearing mice. Whereas treatment with CLS alone or in combination with Dox significantly decreased the tumor content of MDA when compared to either the untreated control (*p* < 0.001 and < 0.01, respectively) or Dox-treated group (*p* < 0.001 and < 0.001, respectively). Moreover, there was no significant difference in GSH content between Dox-treated and untreated control groups. On the other hand, GSH content was significantly higher in CLS-treated groups either alone or combined with Dox than in the untreated control (*p* < 0.001 and < 0.01, respectively) or Dox-treated group (*p* < 0.001 and < 0.001, respectively).

As shown in Fig. 3c, d, Dox treatment also induced a significant increase in tumor content of IL- 6 and IL- 1β compared to the untreated control group (*p* < 0.001 and < 0.01, respectively). In contrast, these inflammatory cytokines in CLS-treated mice were significantly reduced when compared to the untreated control (*p* < 0.001 and < 0.001, respectively) or Dox-treated group (*p* < 0.001 and < 0.001, respectively). Consequently, CLS and Dox co-treatment significantly diminished the tumor tissue inflammation as compared to the Dox mono-treatment group (*p* < 0.001 and < 0.01, respectively).

Consistent with the histopathological observations, *VEGF* gene expression showed a significant increase in mice treated with Dox when compared to the untreated tumor-bearing mice (*p* < 0.01). In addition, CLS-treated groups either alone or when combined with Dox showed a significant decrease in the gene expression of *VEGF* when compared to both the untreated control (*p* < 0.001 and < 0.001, respectively) and Dox-treated (*p* < 0.001 and < 0.001, respectively) tumor-bearing mice (Fig. 3e).

### Effect of treatment with CLS, Dox, and their combination on the gene expression of the oncogenic β-catenin, cyclin-D1, and survivin in the tumor tissue

**Fig. 4:**
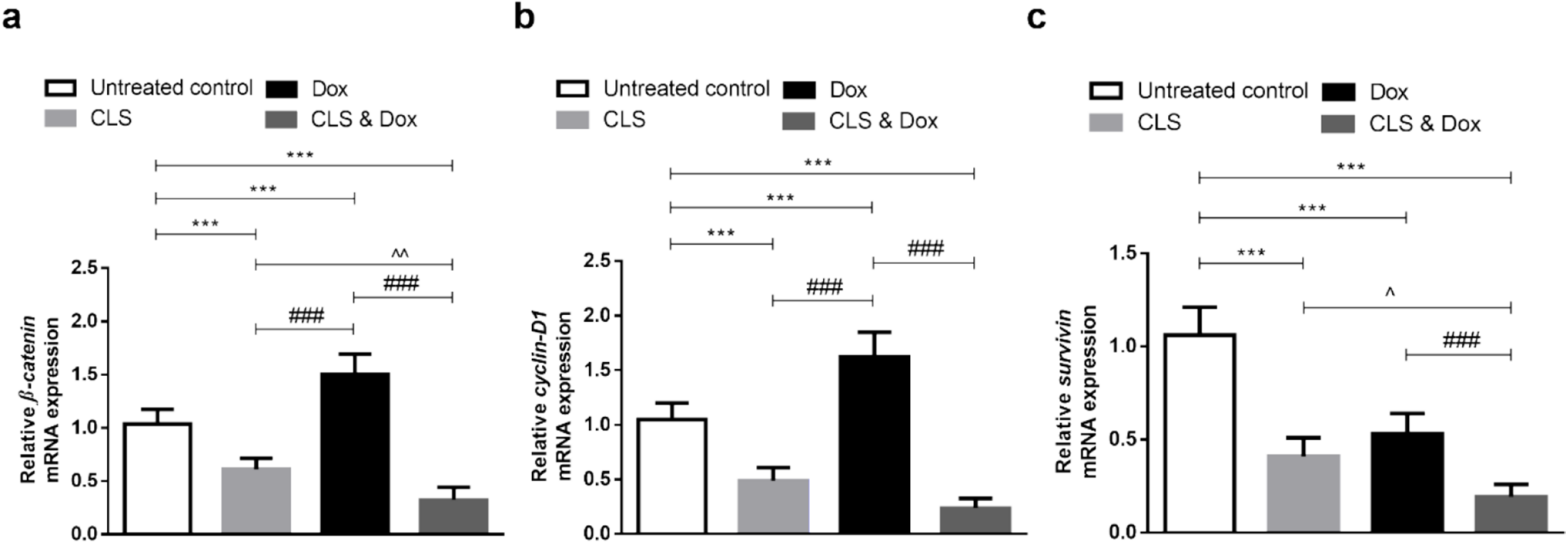
Relative gene expression of (**a**) *β-catenin*, (**b**) *cyclin-D1*, and (**c**) *survivin* in tumor tissue from Ehrlich solid carcinoma (ESC)-bearing mice treated with celastrol (CLS) alone or in combination with doxorubicin (Dox). Results are expressed as Mean ± SD (*n* = 10) and represent expression relative to the *GAPDH* (glyceraldehyde 3-phosphate dehydrogenase) reference gene. Statistical analysis was performed using one-way ANOVA, followed by Tukey's post-hoc test. *** significant at *p* < 0.001 vs. untreated control group; ### significant at *p* < 0.001 vs. Dox-treated group; ^^, ^ significant at *p* < 0.01, *p* < 0.05, respectively vs. CLS-treated group

To estimate the proliferative capacity of tumor cells, relative gene expression of *β-catenin* and *cyclin-D1*, a target gene induced by *β-catenin*, was measured in the Ehrlich tumor of the different experimental groups (Fig. 4a, b). The treatment with Dox was not able to downregulate these genes, causing their overexpression as compared to the untreated control group (*p* < 0.001 and < 0.001, respectively). Whereas CLS alone or in combination with Dox significantly downregulated their expression when compared to the untreated control group (*p* < 0.001 and < 0.001, respectively) and Dox-treated group (*p* < 0.001 and < 0.001, respectively).

*Survivin*, an inhibitor of apoptosis and another targeted gene of *β-catenin*, was also examined (Fig. 4c). A significant decrease in its relative gene expression in all treated tumor-bearing mice with either CLS, Dox, or their combination compared to the untreated control mice (*p* < 0.001, < 0.001, and < 0.001, respectively). Besides, CLS and Dox co-treatment showed a more prominent effect in the reduction of anti-apoptotic protein, *survivin*, than those in mono-treatment groups.

### Effect of treatment with CLS, Dox, and their combination on the protein levels of tumor p53 and caspase- 3

**Fig. 5:**
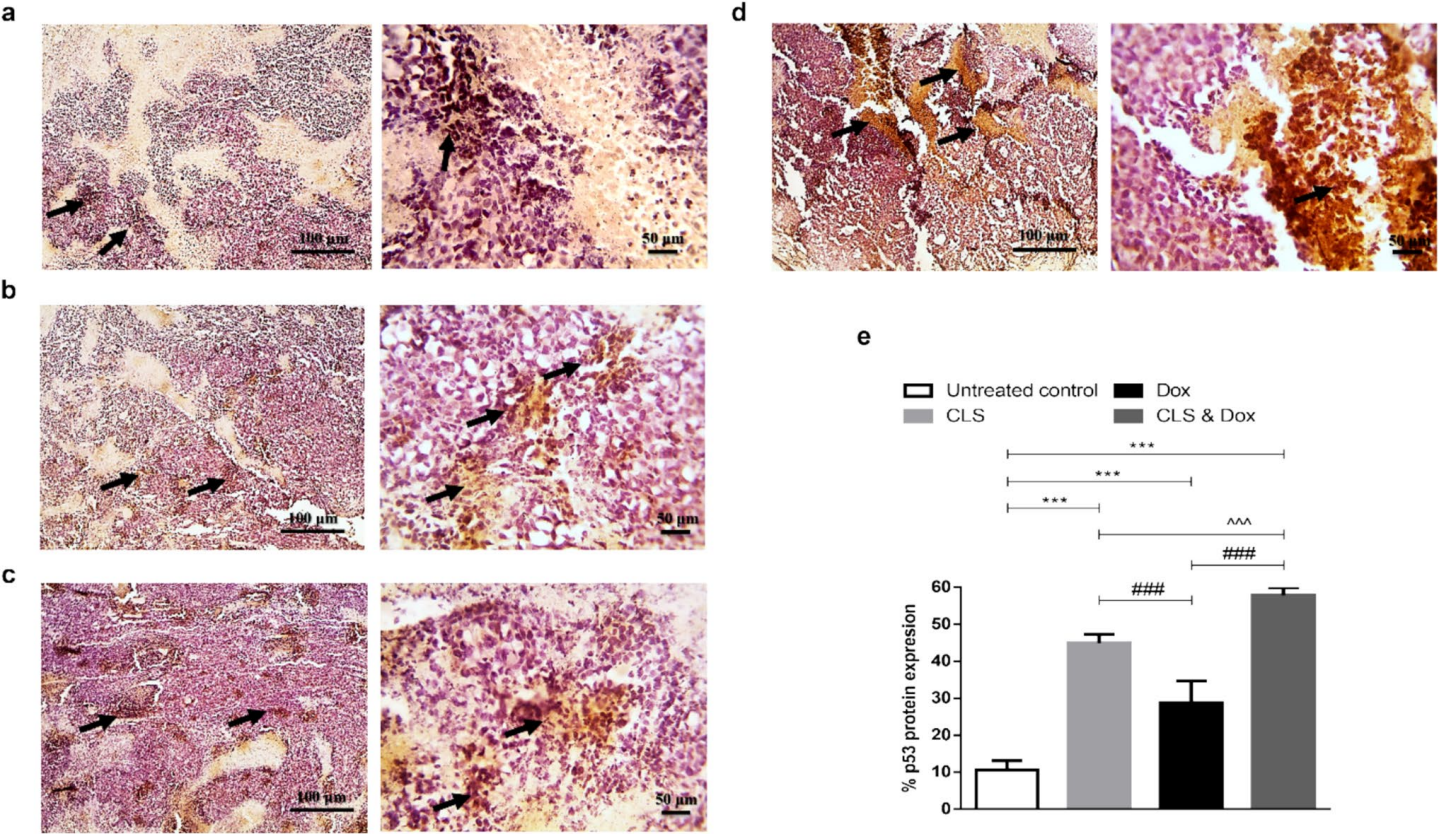
Immunohistochemical staining (IHC) of p53. **a** Untreated control group. **b** Celastrol (CLS)-treated group. **c** Doxorubicin (Dox)-treated group. **d** CLS & Dox-treated group. IHC counterstained with Mayer's hematoxylin. Black arrows point to a positive reaction. Low-magnification (× 100) scale bars = 100 μm and high-magnification (× 400) scale bars = 50 μm. **e** The percentage of p53 protein expression. p53 positive areas were measured using Image J software and expressed as a percentage of the total analyzed area. Results are expressed as Mean ± SD (*n* = 10). Statistical analysis was performed using one-way ANOVA, followed by Tukey's post-hoc test. *** significant at *p* < 0.001 vs. untreated control group; ### significant at *p* < 0.001 vs. Dox-treated group; ^^^ *p* < 0.001 vs. CLS-treated group

**Fig. 6:**
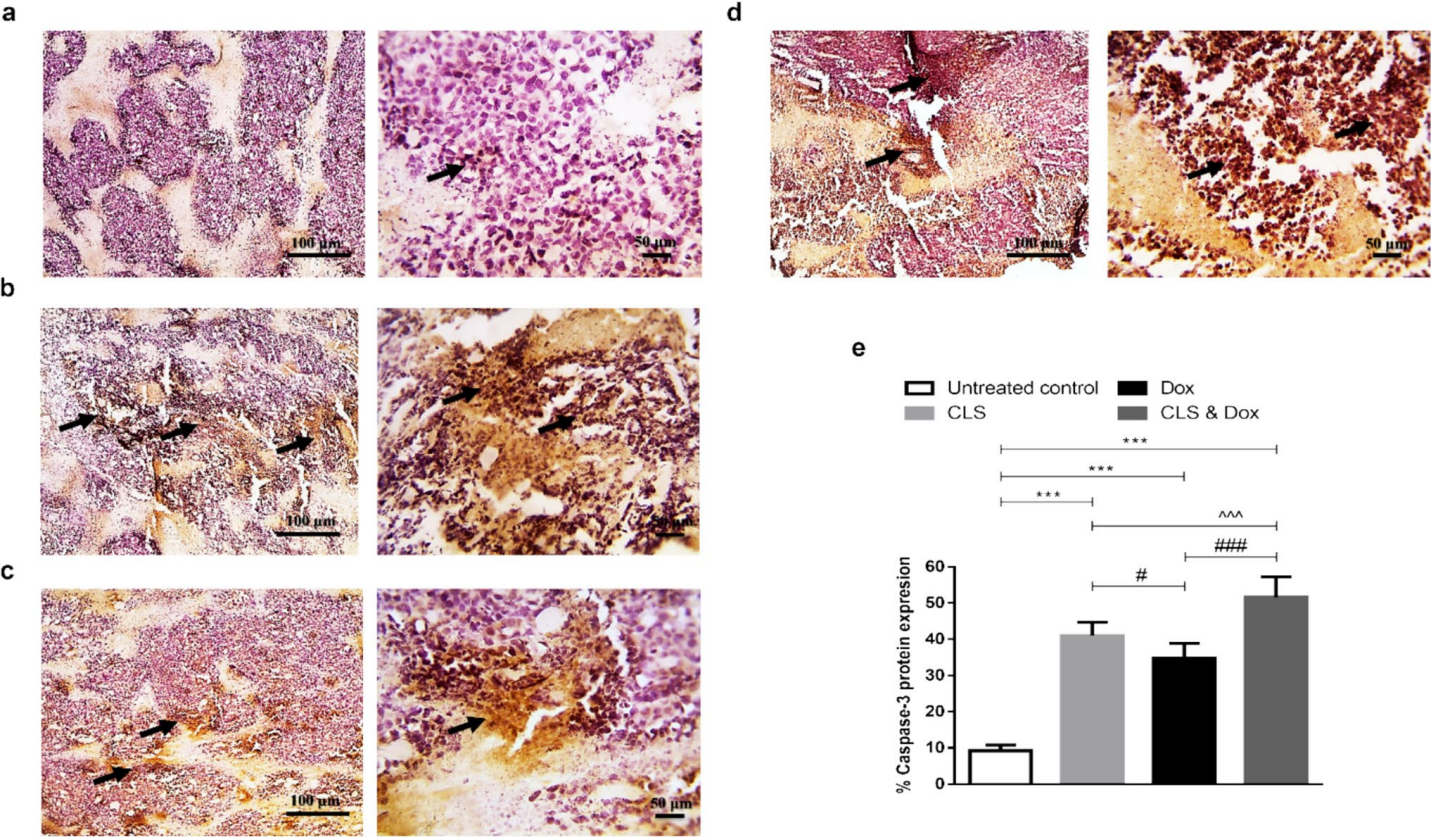
Immunohistochemical staining (IHC) of caspase- 3. **a** Untreated control group. **b** Celastrol (CLS)-treated group. **c** Doxorubicin (Dox)-treated group. **d** CLS & Dox-treated group. IHC counterstained with Mayer's hematoxylin. Black arrows point to a positive reaction. Low-magnification (× 100) scale bars = 100 μm and high-magnification (× 400) scale bars = 50 μm. **e** The percentage of caspase- 3 protein expression. Caspase- 3 positive areas were measured using Image J software and expressed as a percentage of the total analyzed area. Results are expressed as Mean ± SD (*n* = 10). Statistical analysis was performed using one-way ANOVA, followed by Tukey's post-hoc test. *** significant at *p* < 0.001 vs. untreated control group; ###, # significant at *p* < 0.001, *p* < 0.05, respectively vs. Dox-treated group; ^^^ significant at *p* < 0.001 vs. CLS-treated group

The impact of STG and Dox treatment on cell apoptosis was assessed via the intrinsic pathway inducer and the tumor suppressor, p53 (Fig. 5), and its effector, activated caspase- 3 (Fig. 6), by immunohistochemical assessment of the tumor tissues. Stained tumor p53 and caspase- 3 demonstrated low expression in the solid tumor tissue sections dissected from the untreated control mice. In contrast, treatment with either CLS or Dox up-regulated p53 and activated caspase- 3 protein expression in the tumor tissue compared with the control tumor‐bearing group. The combined treatment with CLS and Dox exhibited a more superior apoptotic effect compared to either compound when administered alone, as evidenced by a greater increase in p53 and caspase- 3 protein expression.

## Discussion

CLS exhibits diverse pharmacological effects in a wide range of diseases, including cancer with various underlying mechanisms previously studied (Lim et al. 2021). The purpose of this study is to elucidate its effect on the wnt/β-catenin signaling pathway, which affects cell proliferation, differentiation, and apoptosis, thus exerting crucial roles in tumorigenesis. Besides, the current study investigated combining CLS with Dox, a cytotoxic anthracycline antibiotic widely used in the treatment of various types of cancer, to potentiate its chemotherapeutic effect while reducing some of the associated oxidative imbalance and inflammatory response.

Oxidative stress, an imbalance between the formation of reactive oxygen species and the impaired ability to detoxify these reactive species, reduces the body's antioxidant defenses against angiogenesis and metastasis of tumor cells which encourages the progression of cancer (Arfin et al. 2021; Vona et al. 2021). Treatment of ESC with Dox impaired oxidative balance, as indicated by a significant increase in MDA levels, even when it spared reduced GSH content compared to the untreated tumor-bearing mice. On the other hand, CLS reduced this oxidative imbalance. The antioxidant activity of CLS may be due to its ability to enhance the activity of Nrf2 (nuclear factor erythroid 2-related factor 2), an inducer of various antioxidant enzymes (Sun et al. 2020), which are known to be decreased during Dox treatment (Zhang et al. 2017). Besides, Guan et al*.*, reported that CLS exerted its antioxidant effect in the skeletal muscle of diabetic rats by regulating the AMPK/SIRT3 signaling pathway with a resulting decrease in oxidative stress (Guan et al. 2016). Also, Du et al*.,* found that CLS activates the SIRT3 pathway in human retinal pigment epithelial cells and protects against H_2_O_2_-induced oxidative stress (Du et al. 2019).

Oxidative stress can activate various transcription factors that lead to the expression of several genes involved in the inflammatory pathway. The inflammation triggered by oxidative stress leads to tumor cell survival, proliferation, invasion, and angiogenesis (Reuter et al. 2010). The treatment with Dox has been found to significantly increase the tumor levels of IL- 6 and IL- 1β. While CLS significantly decreased both when compared to the untreated ESC-bearing mice. This is in line with Wang et al*.,* who attributed the anti‐inflammatory effect of CLS and downregulation of these inflammatory cytokines to its activation of AMPK/SIRT3 pathway (Wang et al. 2020). You et al*.,* also found that CLS downregulates IL- 1β through the inhibition of the MEK/ERK signaling pathway (You et al. 2021). Besides, CLS inhibits both IκBα phosphorylation and subsequent NF-κB activation by preventing TAK1 (TGF-β-activated kinase 1) phosphorylation. NF-κB inhibition often downregulates the expression of pro-inflammatory mediators like IL- 6 and IL- 1β (Xu et al. 2021; Zhang et al. 2021). Whereas Guo et al*.,* Hajra et al*.,* and Arunachalam et al*.,* found that Dox induces the p38 MAPK/NF-κB pathway controlling the production of downstream pro-inflammatory cytokines, including IL- 6 and IL- 1β (Guo et al. 2013; Hajra et al. 2018; Arunachalam et al. 2021).

Tumor-associated angiogenesis is necessary for the growth and metastasis of solid tumors and an important part of controlling cancer progression (Haibe et al. 2020). Several angiogenic factors, such as growth factors and cytokines have been described to control angiogenesis. Of those, VEGF is a major regulator of angiogenesis (Fallah et al. 2019). Treatment with Dox significantly increased *VEGF* gene expression and subsequent neo-angiogenesis. However, CLS has been found to inhibit tumor angiogenesis and significantly decrease *VEGF* gene expression as compared to the untreated ESC-bearing mice. The inhibitory effect of CLS on angiogenesis is mediated mainly by the suppression of HIF- 1α/VEGF signaling (Li et al. 2020; Fang and Chang 2021). CLS can also target another important transcription factor, STAT3 (signal transducer and activator of transcription- 3), which has been implicated in tumor progression (Loh et al. 2019; Garg et al. 2021). CLS inhibition of STAT3 has been mediated by the inhibited activation of upstream JAK1/2 (Janus kinase1/2). This inhibition is postulated to mediate NF-κB inhibition, leading to inhibited expression of *VEGF* (Rajendran et al. 2012). Additionally, since the IL- 6 is a major inducer of STAT3 phosphorylation (Johnson et al. 2018), the present study suggests that CLS-induced reduction of IL- 6 may also suppress the STAT3 signaling pathway, which in turn downregulated *VEGF*.

The aggressiveness of various neoplasms increases with uncontrolled proliferation and a parallel increase in the Wnt/β-catenin signaling, a key pathway in multiple aspects of cellular processes, including cell proliferation, differentiation, and morphogenesis (Azbazdar et al. 2021). β-catenin regulates the expression of a significant number of oncogenes, such as *cyclin-D1*, which is an important checkpoint regulator that allows G_1_ phase progression (Lecarpentier et al. 2019). A significant decrease in the expression of *β-catenin* and *cyclin-D1* has been found in CLS-treated mice as compared to the untreated tumor-bearing mice. CLS has been found to induce ubiquitin–proteasome degradation of β-catenin via phosphorylation of YAP (Yes-associated protein), a major downstream effector of the Hippo pathway, and also dependent on activating the LKB1/AMPKα pathway, partially through the heat shock factor 1 (HSF1) (Wang et al. 2019). The degradation of β-catenin downregulates the transcription of *cyclin-D1* and ultimately arrests the tumor cells at the G_1_/G_0_ phase (Shang et al. 2017). CLS also downregulates *cyclin-D1* through the suppression of IL- 6-induced phosphorylation of STAT3 (Kannaiyan et al. 2011), and TAK1-mediated NF-κB activation (Sethi et al. 2007).

The primary mechanism, by which cancer cells thrive, is to avoid apoptosis; thus, effective anticancer treatments would stimulate the apoptotic pathways to eradicate cancer cells (Pfeffer and Singh 2018). In the present study, the treatment with CLS remarkably upregulate the expression of pro-apoptotic proteins, p53 and caspase- 3, while downregule the gene expression of the anti-apoptotic protein, *survivin*. This agrees with the study of Zhu and Wei. As they found that CLS induced upregulation of caspase- 3 while downregulated survivin and attributed its apoptotic induction to the blockage of PI3 K/Akt‐mediated signaling by upregulating PTEN (Zhu and Wei 2020). As well, Shen et al*.,* found that CLS induces caspase-dependent apoptosis by inhibiting the activation of mTOR (mammalian target of rapamycin) (Shen et al. 2021). Since survivin is regulated by β-catenin (Boidot et al. 2014), the current study proposes that CLS induces apoptosis via downregulating β-catenin. Besides, survivin is another target gene product regulated by STAT3 and NF-κB, both of which are suppressed via CLS (Sethi et al. 2007; Kannaiyan et al. 2011; Rajendran et al. 2012).

## Conclusions

Based on our experimental results, we conclude that CLS dampens tumor cell proliferation as well as induces apoptosis and cell cycle arrest by targeting the wnt/β-catenin signaling pathway. The study also opens a new perspective for combining CLS with Dox to enhance its chemotherapeutic effects and reduce the oxidative imbalance and inflammatory responses associated with Dox treatment.

## Data Availability

All source data for this work (or generated in this study) are available upon reasonable request.

## Abbreviations

ESC: Ehrlich solid carcinoma
CLS: Celastrol
Dox: Doxorubicin
MDA: Malondialdehyde
GSH: Glutathione
IL- 6: Interleukin 6
IL- 1β: Interleukin 1β
VEGF: Vascular endothelial growth factor
